# Comparative analysis of lateral maxillary sinus augmentation with a xenogeneic bone substitute material in combination with piezosurgical preparation and bony wall repositioning or rotary instrumentation and membrane coverage: a prospective randomized clinical and histological study

**DOI:** 10.1007/s00784-022-04494-x

**Published:** 2022-05-20

**Authors:** Bálint Molnár, Anne-Kathrin Jung, Zsuzsanna Papp, Anna Martin, Kristóf Orbán, Annica Pröhl, Ole Jung, Mike Barbeck, Péter Windisch

**Affiliations:** 1 Department of Periodontology, Semmelweis University Budapest, Szentkiralyi u. 47, 1088 Budapest, Hungary; 2 Clinic and Policlinic for Dermatology and Venereology, University Medical Center Rostock, Rostock, Germany; 3 BerlinAnalytix GmbH, Berlin, Germany

**Keywords:** Maxillary sinus floor augmentation, Piezoelectric surgery, Bony wall repositioning, Collagen membrane, Xenografts, Dental implant

## Abstract

**Objectives:**

The present randomized controlled clinical study aimed to investigate if, in lateral maxillary sinus augmentation, the repositioned bony wall or the application of a collagen membrane results in more preferable new hard tissue formation.

**Materials and methods:**

Forty patients were divided into two study groups. Both groups received a xenogeneic bone substitute material (BSM) during lateral sinus augmentation. In the bony wall group (BW), following piezosurgery, the retrieved bony wall was repositioned. In the collagen membrane group (CM), following rotary instrument preparation, collagen membrane coverage was applied. After 6 months, biopsies were taken to histologically analyze the percentage of BSM, connective tissue (CT), and newly formed bone (NFB) following both approaches.

**Results:**

Forty implants were placed and 29 harvested biopsies could be evaluated. Duration of surgery, membrane perforations, and VAS were detected. Histomorphometrical analysis revealed comparable amounts of all analyzed parameters in both groups in descending order: CT (BW: 39.2 ± 9%, CM: 37,9 ± 8.5%) > BSM (BW: 32.9 ± 6.3%, CM: 31.8 ± 8.8%) > NB (BW: 27.8 ± 11.2%, CM: 30.3 ± 4.5%).

**Conclusions:**

The results of the present study show that the closure of the access window by means of the retrieved bony wall or a native collagen membrane led to comparable bone augmentation results.

**Clinical trial:**

clinicaltrials.gov NCT04811768.

**Clinical relevance:**

Lateral maxillary sinus augmentation with the application of a xenogeneic BSM in combination with a native collagen membrane for bony window coverage represents a reliable method for surgical reconstruction of the posterior maxilla. Piezosurgery with bony window repositioning delivers comparable outcomes without membrane coverage.

## Introduction

In dentistry, the augmentation of the bone within the maxillary sinus is an important as well as a common procedure for dental implant placement [1–5]. Various techniques are available, including the lateral window technique, crestal access, and vertical osteotome-mediated sinus floor elevation. Crestal access is indicated in cases with at least 6 mm of subantral vertical bone height [1, 3, 6–8]. The lateral approach is a clinically significant surgical procedure for hard tissue reconstruction allowing for reliable clinical results in implant dentistry and enabling implant placement even in pneumatized sinus conditions of the posterior maxilla in cases with less than 6 mm of subantral vertical bone height. Implant placement may be done simultaneously or delayed to the lateral approach [9–16]. It was initially performed by Dr. Hilt Tatum Jr. in the 1970s, followed by the first publications of Boyne and James in the 1980s [17]. Conventionally, the lateral approach is performed with a full-thickness mucoperiosteal flap; nevertheless, according to a recent study, the split-thickness flap design may represent a more favorable clinical strategy to avoid significant postoperative blood supply disturbances in the maxillary vestibule [18]. Therefore, it may be also applicable for lateral sinus floor elevation. The lateral approach can be carried out using flap techniques with different types of releasing incisions (e.g., quadratic, trapezoid) in combination with different bone-grafting materials, e.g.: autologous bone or allogeneic, xenogeneic, and synthetic bone substitute materials [1, 6, 10, 16, 19–24].

In recent years, different aspects of this surgical technique have been investigated regarding dental instruments, bone substitute materials (BSM), and the necessity to reposition the bony wall. Thereby, different studies showed favorable results when piezoelectric surgery was used instead of conventional rotary instruments in terms of perforation of the Schneiderian membrane or to reposition the minimally invasively retrieved sinus bony wall [12, 15, 16, 19, 25–32]. Most studies reported satisfactory results when the bony wall was repositioned into the lateral window, regardless of the used BSM, acting as an autologous barrier for bone growth such as an autologous bone transplant [11, 15, 23, 33]. However, collagen membranes are used by a broad number of clinicians for coverage of the bony window as it has been stated that such kind of biomaterial allows for the prevention of soft tissue invasion of the sinus [2, 21, 34]. Additionally, it has been reported that the application of membranes may allow for a greater amount of bone regeneration within the sinus cavity [2, 34]. However, there are only a few studies comparing the application of resorbable collagen membranes to cover the bony window instead of bony wall repositioning with regard to complication rate, duration of surgery, and patient morbidity [22, 33, 35]. Moreover, data is lacking regarding the comparison of bony wall repositioning to collagen membrane coverage of the lateral sinus window in combination with a natural BSM in a clinical study. Furthermore, histologic and histomorphometric data of the healing or integration events of either of these methods have not been reported yet. Thus, the present study aimed to evaluate the efficacy of piezosurgically retrieved lateral bony wall repositioning in lateral maxillary sinus floor augmentation compared to the application of a resorbable collagen membrane in combination with a xenogeneic BSM in a prospective randomized controlled clinical and histological study.

## Materials and methods

### Study groups and study design

In total, 40 patients between the ages of 18 and 70 and a need for maxillary sinus floor augmentation using the lateral approach were enrolled in the study at the Department of Periodontology, Semmelweis University, Budapest, Hungary. The study protocol was approved by the Semmelweis University Regional and Institutional Committee of Science and Research Ethics (Approval Number SE TUKEB 7/2017). Surgical interventions were undertaken with the understanding and written informed consent of each subject. The patients were treated in full accordance with ethical principles, including the World Medical Association Declaration of Helsinki [version 2008]. The study was registered at clinicaltrials.gov (NCT04811768 Unique Protocol ID: Sinus-Semmelweis-Perio). Inclusion criteria were as follows: At least one missing maxillary premolar or molar with at least 7 mm crestal bone width and maximally 5 mm residual bone height at the sinus floor confirmed by preoperative cone-beam tomography [CBCT]. Patients with edentulous areas located posterior to the remaining natural teeth as well patients with single tooth gaps were recruited. Full mouth plaque and bleeding scores (FMPS and FMBS) < 20% as well as satisfactory patient compliance (e.g., to participate in follow-up procedures). Baseline subject characteristics are summarized in Table 1. Exclusion criteria were as follows: smoking, clinically relevant diseases (e.g., diabetes, rheumatism cancer), untreated periodontitis, systemic steroid or bisphosphonate use, acute or chronic inflammatory processes, previous endoscopic sinus surgery or sinus floor elevation, GBT/GTR-treatment at the study site, and tooth removal within 6 weeks prior to surgery. All clinical and radiographic parameters were ascertained by an experienced examiner in order to check the eligibility of each patient for the study. Patients were randomly divided into two groups using a computer-generated randomization scheme. Bony wall (BW) group (*n* = 20): lateral sinus floor augmentation with BSM (cerabone, botiss biomaterials GmbH, Zossen, Germany) using NSK VarioSurg3 piezoelectric device (NSK Europe GmbH, Eschborn, Germany), bony window covered by lateral bony wall repositioning. Collagen membrane (CM) group (*n* = 20): lateral sinus floor augmentation with BSM using conventional rotary instruments, bony window covered by a resorbable collagen membrane (collprotect membrane, botiss biomaterials GmbH, Zossen, Germany).

**Table 1:** Baseline subject characteristics

	Test group	Control group
Age (years)	51	48
SD	9	8
FMPS (percentage)	15	14
SD	4	4
FMBS (percentage)	17	15
SD	2	3

### Surgical technique

**Fig. 1:**
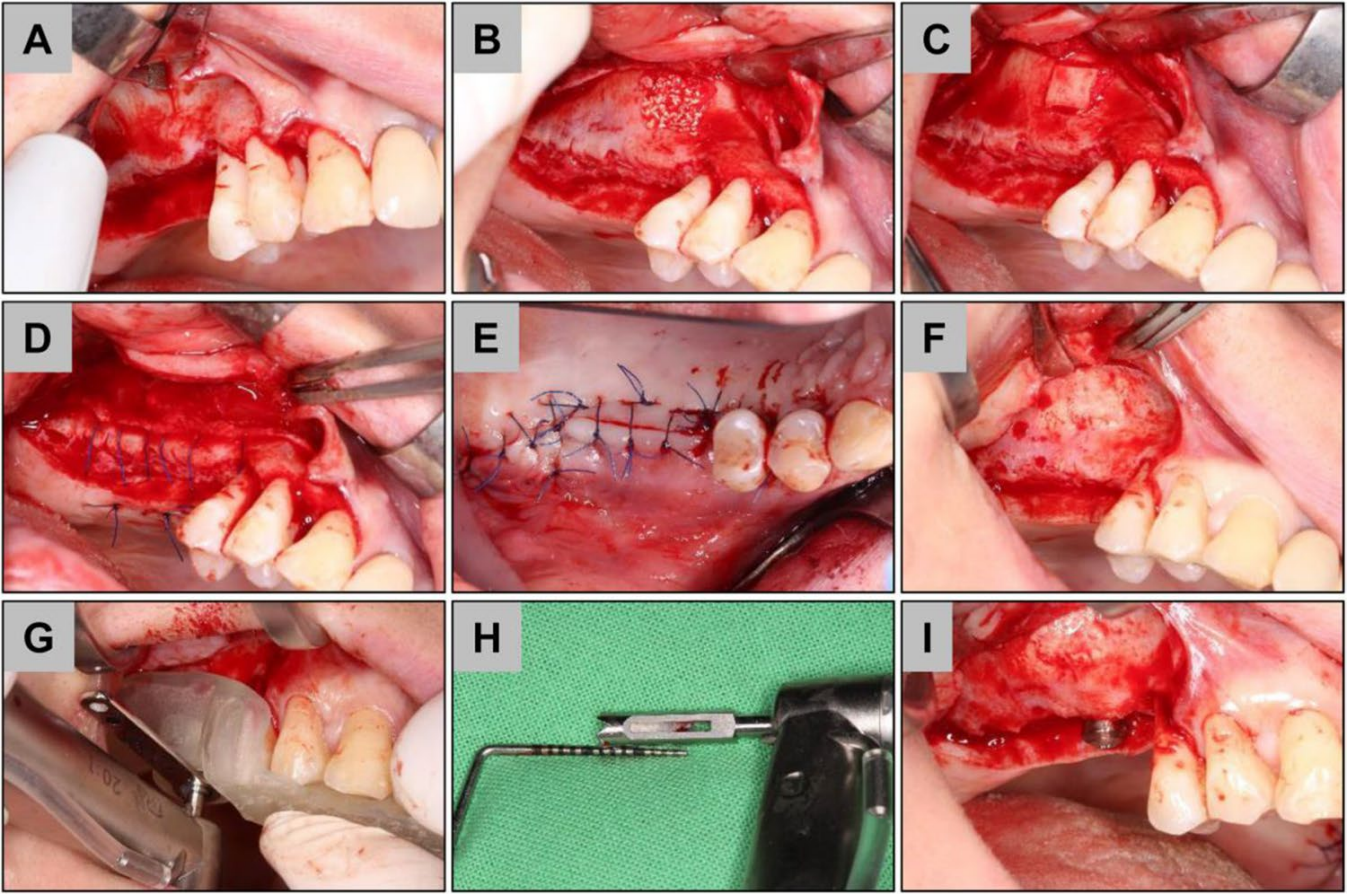
Exemplary images of the implantation procedure in the bony wall (BW) group. **A** Piezoelectric window preparation, **B** cerabone insertion, **C** bony wall repositioning, **D** periosteal sutures, **E** mucosal sutures, **F** 6 months reentry, **G** guided biopsy harvesting, **H** core biopsy, and **I** implant insertion

**Fig. 2:**
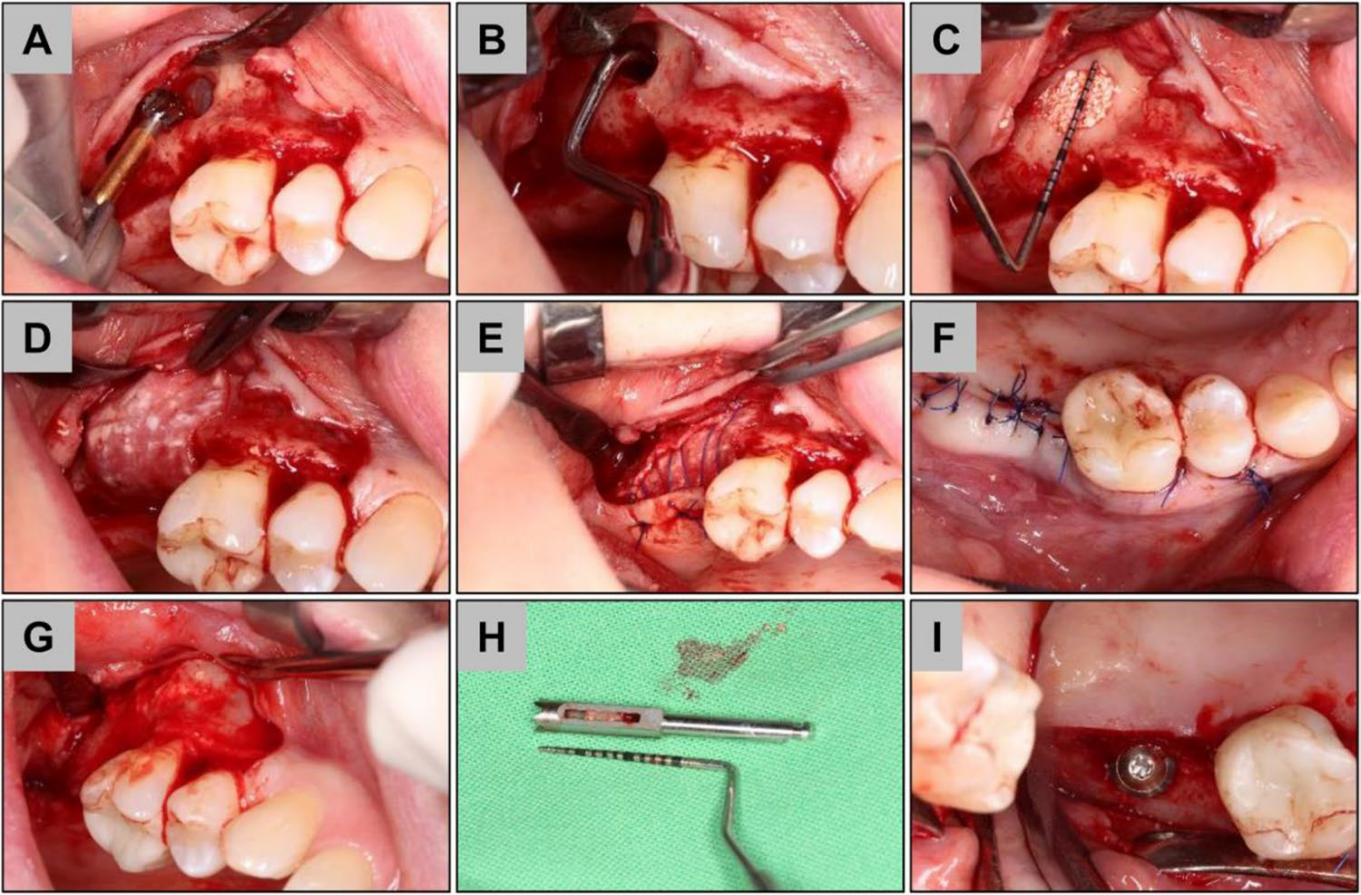
Exemplary images of the implantation procedure in the collagen membrane (CM) group. **A** Rotary window preparation, **B** sinus mucosa elevation, **C** cerabone insertion, **D** collprotect membrane coverage, **E** periosteal sutures, **F** mucosal sutures, **G** 6 months reentry, **H** core biopsy, and **I** implant insertion

Prior to surgical intervention, supra- and subgingival scaling, root planning, and polishing were performed, and oral hygiene instructions were given to every patient. CBCT planning was performed (Planmeca Oy, Helsinki, Finland) and 3D guided surgery stents (Dicomlab, Szeged, Hungary) were fabricated prior to surgery for optimal implant positioning and mapping of window preparation during surgery. Operations were performed under local anesthesia. The elevation of the mucoperiosteal flap was achieved after a midcrestal and a single mesial vertical incision followed by split-thickness flap preparation (Figs. 1 and 2).

In the BW group, piezosurgery (NSK Variosurg3, NSK Europe GmbH, Eschborn, Germany) was used to prepare a 7 by 7 mm quadratic bony window 5 mm apically from the bony crest and was lifted outward (Fig. 1). The position of the bony window was mapped via prefabricated 3D guided stents according to the previously planned implant position. The sinus membrane was elevated using hand instruments to allow bone grafting. In the CM group, a 7 by 7 mm quadratic bony window preparation was carried out using 3 mm diameter rotary diamond burs at 400 RPM and the sinus membrane was elevated by hand instruments (Fig. 1). The bony wall was completely eliminated during the process. Bone grafting was performed using a particulate bovine xenograft with grain size between 0.5—1.0 mm (Fig. 1). In the BW group, the bony wall was repositioned (Fig. 1). In the CM group, a resorbable porcine collagen membrane (collprotect membrane, botiss biomaterials GmbH, Zossen, Germany) was applied for lateral window coverage (Fig. 2). Wound margins were adapted in two layers by horizontal mattress and single interrupted non-resorbable monofilament sutures (Dafilon 4/0 and 5/0, Braun Melsungen AG, Tuttlingen, Germany) (Figs. 1 and 2). Sutures were removed after 14 days postoperatively. For implant placement and biopsy removal, re-entry surgery was performed after 6 months with the same flap design as described above. From a crestal approach, at the predefined implant positions, core biopsies were retrieved by 3.2 mm outer/2.6 mm inner diameter trephine burs (Komet Dental, Lamgo, Germany) in all cases of both test and control groups. Subsequently, 4.1 mm diameter, 10 mm dental implants (Straumann Tissue Level 4.1/10 mm RN; Straumann, Basel, Switzerland) were placed at previously augmented sites by means of guided surgery. Following all stages, patients received postoperative antibiotic therapy (amoxicillin + clavulanic acid 3 × 625 mg for 7 days, in case of allergy, clindamycin 3 × 300 mg for 7 days) and pain relief medication (diclofenac max. 3 × 50 mg according to patients’ needs). Local chemoprophylaxis (0.2% chlorhexidine mouth rinse) was used twice a day for 2 weeks and patients were forbidden dental cleaning at the operation side for two weeks.

### Clinical evaluation

Each surgery was performed by the same operator [BM]. The primary outcome measure was the total duration of sinus floor augmentation surgery. Secondary clinical outcome measures were duration of lateral window preparation and duration of sinus mucosa preparation; subjective patients’ discomfort, assessed by a visual analogue score [VAS], recorded on the day of the surgery, 1, 2, and 3 days following maxillary sinus augmentation; postoperative hematoma; and swelling evaluated by an investigator [ZSP] on an arbitrary scale of 0 to 3.

### Histological preparation and histomorphometry

Percentage of newly formed bone, bone substitute, and connective tissue assessed by histomorphometry were further secondary outcome measures. Core biopsies were immediately fixed in 4% formalin for 24 h before their transfer to phosphate-buffered saline [PBS] for further histologic workup. All biopsies were dehydrated in a series of increasing alcohol concentrations before paraffin embedding and sectioning of 3–5 µm slides by means of a rotation microtome (Cut 6062, SLEE medical GmbH, Mainz, Germany). These slides were stained by hematoxylin–eosin (HE), Masson trichrome, and von Kossa for histologic analysis with special attention to newly formed bone (NFB), residual bone substitute material (BSM), and connective tissue (CT).

**Fig. 3:**
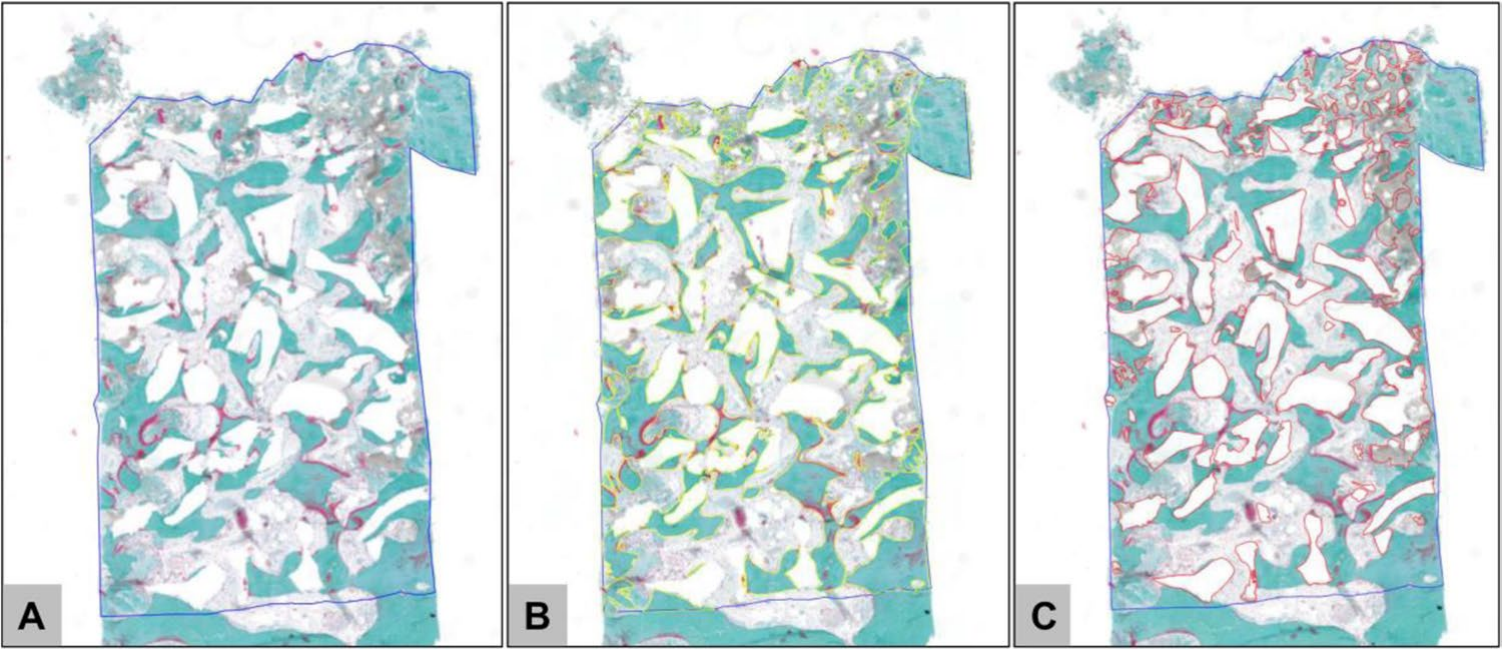
Exemplary total scans of the analyzed sinus biopsies and the histomorphometrical measurements. **A** Marking of the total implant area (blue line) within a sinus biopsy. **B** Marking of the fraction of new bone formation (yellow lines). **C** Marking of the remaining bone substitute fraction (red lines) (Masson Goldner-staining, × 100 magnification)

Although 40 implants were placed, only 29 harvested biopsies could be evaluated due to problems with the histological workup in the case of 11 biopsies, which restrict both the histological and the histomorphometric analysis. Histological analysis was performed by two independent investigators (AJ and MB) focused on the cell responses and the integration behaviors. Images were recorded using a light microscope (Axiscope 40, Carl Zeiss, Oberkochen, Germany) combined with a digital camera (Axiocam 105 color, Carl Zeiss, Oberkochen, Germany), and the Zen software (version 2.3, blue edition, Carl Zeiss, Oberkochen, Germany). Histomorphometry was achieved by initial digitalization of the slides using a light microscope (Axiscope 40, Carl Zeiss, Oberkochen, Germany) connected with a scanning table (EK 14 mot, Märzhäuser, Wetzlar, Germany), a digital camera (AxioCam MRc 5, Carl Zeiss, Oberkochen, Germany), and Zeiss AxioCam software (Axio Vs40, version 4.8.2.0, Carl Zeiss, Oberkochen, Germany) at × 10 magnification. Finally, the Zen software was used to determine the total implant area (TIA) as well as the different fractions, i.e., NFB, BSM, and CT (Fig. 3). NFB, BSM, and CT were set in relation to TIA to get percentage values that allowed for statistical comparison of both study groups.

### Statistical analysis

Treatment durations were statistically analyzed by Paired t-test for group statistics. Levene’s test for equality of variances and t-test for equality of means were applied as independent samples test. The frequency of sinus membrane perforations was statistically assessed by the chi-squared test. Quantitative histological data were statistically analyzed by analysis of variance (ANOVA) with Brown-Forsythe evaluation using GraphPad Prism 8.0.2 (GraphPad Software, San Diego, CA, USA). Statistical differences were designated as significant if *p*-values were less than 0.05 (* *p* ≤ 0.05) and highly significant if *p*-values were less than 0.01 (** *p* ≤ 0.01). Finally, the data were shown as mean ± standard deviation.

## Results

### Clinical results

All 40 sinus floor augmentation surgeries were successful, significant arterial injury or intraoperative bleeding did not occur, and the healing period was uneventful in each case. In the BW group, the duration of surgery averaged 45.8 ± 11.3 min compared to 49.2 ± 11.3 min in the CM group. The mean duration of lateral sinus window preparation was 4.5 ± 1.4 min in the BW group compared to 5.9 ± 3.7 min in the CM group. The mean duration of sinus mucosa preparation was 5.8 ± 3.3 min in the BW group compared to 7.2 ± 4.3 min in the CM group (Table 2). The number of sinus membrane perforations was 6 out of 20 in the BW group, compared to 7 out of 20 in the CM group. Postoperative discomfort was evaluated using VAS. In the BW group vs. CM group, on the day of the surgery, 1, 2, and 3 days postoperatively, mean VAS measured 30.9 ± 31.3 vs. 44.5 ± 27.1, 19.8 ± 26.1 vs. 29.4 ± 26.6, 12.9 ± 20.3 vs. 24.8 ± 23.2, and 9.5 ± 16.5 vs. 17.6 ± 21.1, respectively. In the BW group vs. CM group, 3 days postoperatively, mean postoperative hematoma score measured 0.7 ± 0.9 vs. 1.3 ± 1.3. In the BW group vs. CM group, 3 days postoperatively, mean postoperative edema score measured 1.9 ± 1.0 vs. 1.8 ± 0.8 (Table 3). None of the differences was statistically significant (*p* > 0.05). The amount of newly formed hard tissues was sufficient for implant placement in all cases as confirmed by 6 months CBCT scans. At 6 months, reentry surgery was performed. At least 7 mm ridge width was detected at re-entry, additional grafting was not necessary in any of the cases. In the BW group, complete reintegration of the repositioned bony wall was observed in all cases. In the CM group, BSM particles embedded in native bone were observed in the area of the lateral window. A total of 40 implants (Straumann tissue level 4.1/10 mm RN; Straumann, Basel, Switzerland) were placed at sites previously treated with maxillary sinus augmentation. Three months later, implants were uncovered and restored with screw-retained fixed partial dentures.

**Table 2:** Surgical time

	Test group	Control group
Duration of Surgery (min)	45.8	49.2
SD	11.3	11.3
Duration of sinus mucosa preparation (min)	5.8	7.2
SD	3.3	4.3
Duration of window preparation (min)	4.5	5.9
SD	1.4	3.7

**Table 3:** Postoperative pain (VAS), hematoma, and edema

	Test group	Control group
VAS—0 days	30.9	44.5
SD	31.3	27.1
VAS—1 day	19.8	29.4
SD	26.1	26.6
VAS—2 day	12.9	24.8
SD	20.3	23.2
VAS—3 day	9.5	17.6
SD	16.5	21.1
Hematoma	0.7	1.3
SD	0.9	1.3
Edema	1.9	1.8
SD	1.0	0.8

### Histological results

All patients participated throughout the study. In total, 29 patient biopsies out of 40 harvested core biopsies could be analyzed. Thirteen biopsies from the BW group and 16 biopsies from the CM group were evaluable. Eleven biopsies were discarded because of a lack of measurable material or difficulties to process the biopsies.

**Fig. 4:**
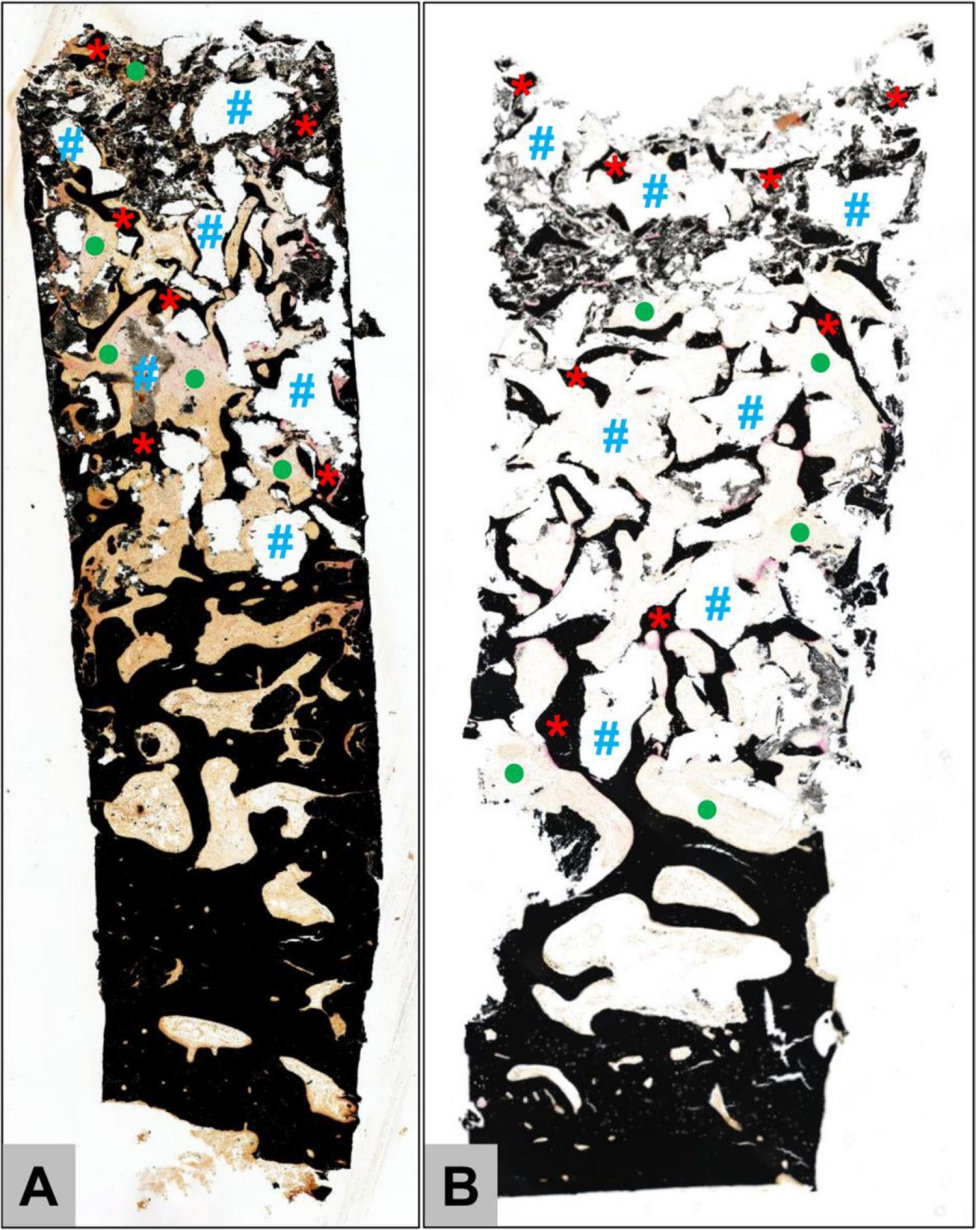
Exemplary overviews of biopsies form **A** the bony wall (BW) group and **B** the collagen membrane [CM] group. In both groups, newly formed bone tissue (red asterisks) was grown throughout the complete implantation area of the xenogeneic bone substitute (blue hashes). The tissue distribution, i.e., the amounts of newly formed bone, remaining bone substitute, and connective tissue (green points) were comparable (“total scan,” von Kossa staining, × 100 magnifications)

**Fig. 5:**
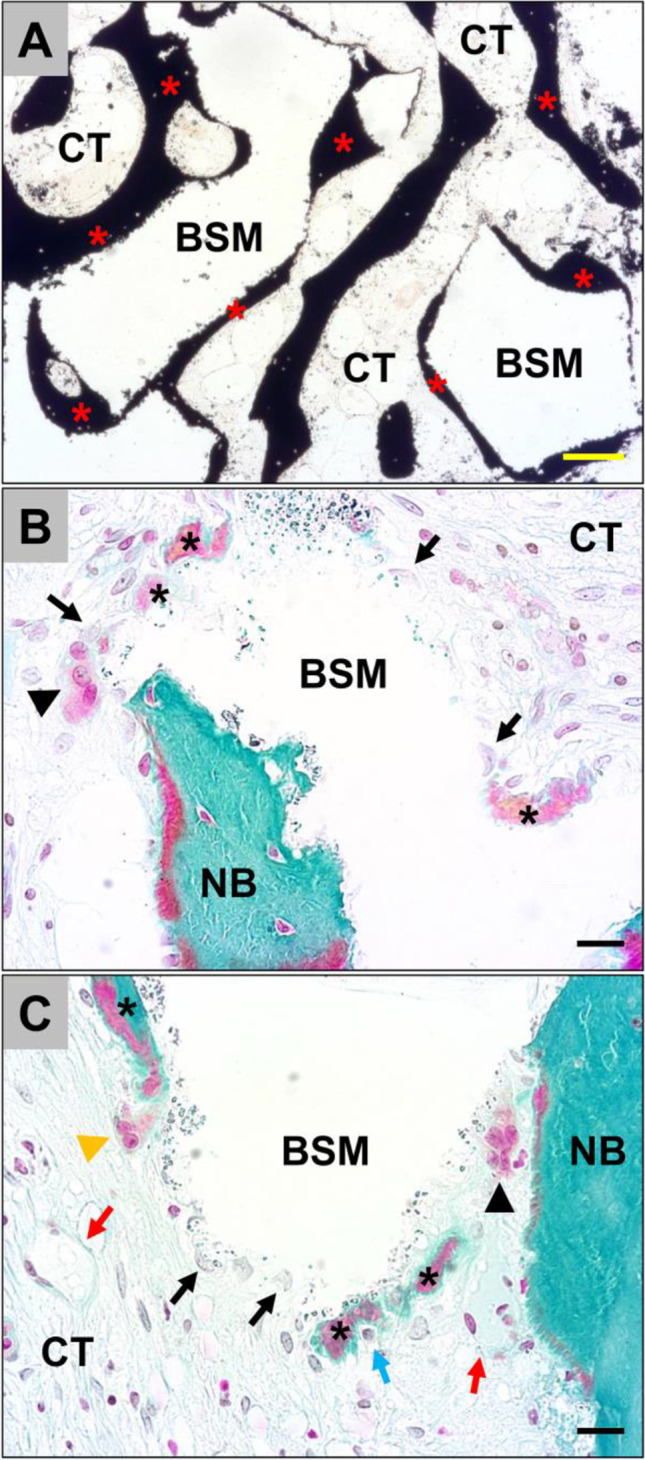
Exemplary histological images from the collagen membrane (CM) group showing the integration behavior of the xenogeneic bone substitute material (BSM) that was observed in both study groups without any differences. **A** The material granules were mainly embedded within newly grown bone matrix (red asterisks). CT = connective tissue (von Kossa staining, × 100 magnification, scalebar = 50 µm). **B** and **C** New bone formation (black asterisks) was regularly observed at the BSM surfaces associated with active osteoblasts (blue arrow in C) indicating that the bone growth process was still in process. At the surface areas that were covered by connective tissue (CT) mainly macrophages (black arrows) and single multinucleated giant cells (black arrowhead) were detected. Interestingly, osteoclasts (yellow arrowhead) were regularly found in direct neighborhood of these areas and their cellular arrangement did not significantly differ from the material-associated giant cells. NB = newly formed bone tissue, blood vessels = red arrows (Masson Goldner staining, × 400 magnifications, scale bars = 10 µm)

The histological analysis showed that similar tissue reactions and integration pattern of the xenogeneic BSM were observable in both the BW and CM groups. Thus, newly formed bone was found mostly attached to the surfaces of the BSM granule surfaces throughout the whole implantation area in both groups (Fig. 4). The histological analysis furthermore showed that the tissue reactions to the granules of the xenogeneic BSM were comparable (Fig. 5). Most of the surface areas of the BSM granules were covered by newly formed bone tissue (Fig. 5A). Within the areas that were covered by connective tissue most often small borders of new bone matrix associated with osteoblasts, i.e., multinucleated cells associated with the bone matrix, were present indicating that the bone growth process was not completed (Fig. 5B and C).

Additionally, macrophages and single multinucleated giant cells, i.e., cells associated with the BSM, were observable in these areas, while the intergranular connective tissue showed no signs of inflammatory processes (Fig. 5B and C).

### Histomorphometric results

**Fig. 6:**
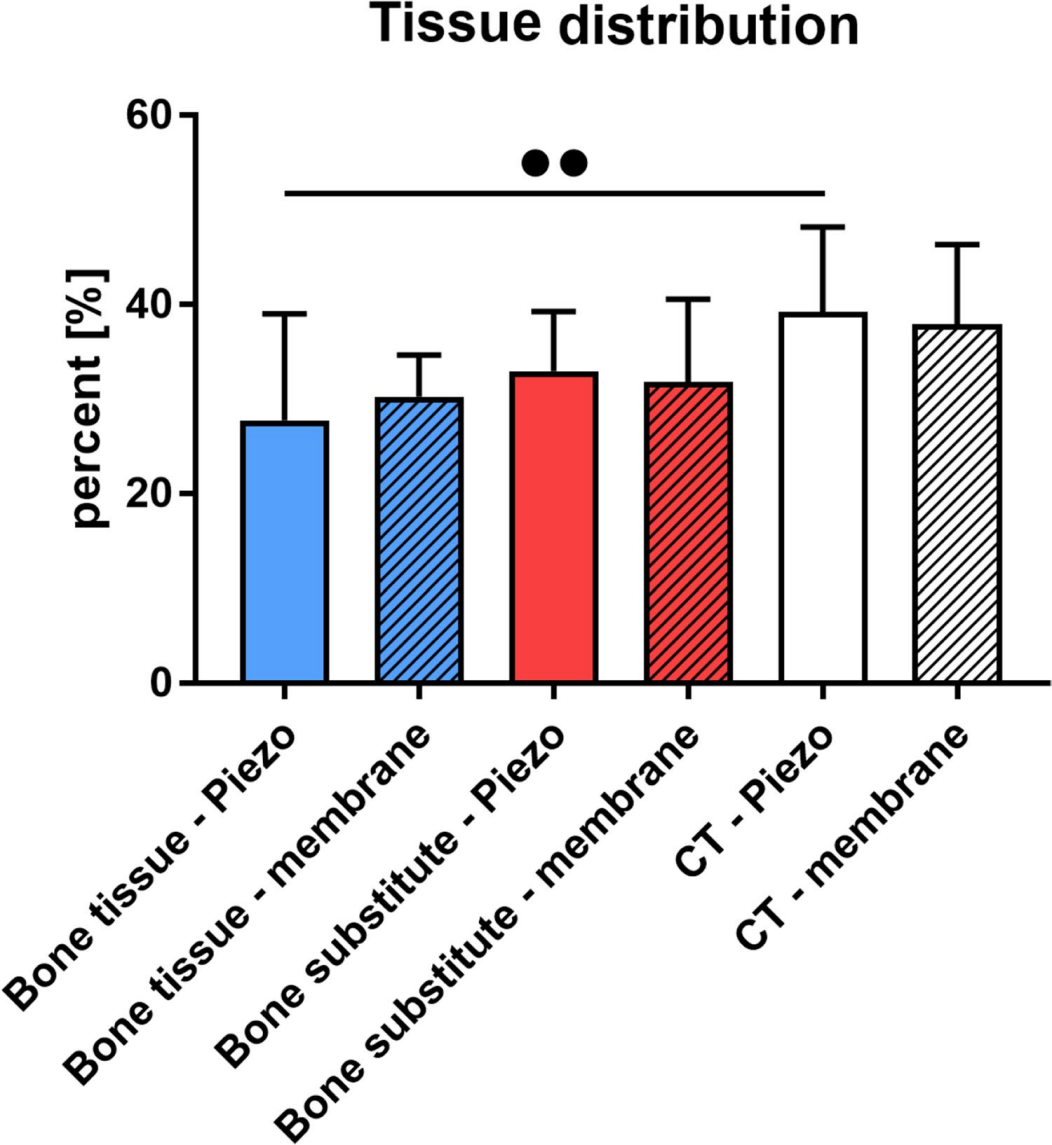
Results of the histomorphometrically measured tissue distribution (** p < 0.01). BW: bony wall group, CM: collagen membrane group

The histomorphometric analysis showed that the tissue fractions in both study groups were comparable without any interindividual differences (Fig. 6). Thus, amounts of newly formed bone showed mean values of 27.8 ± 11.2% in the BW group and 30.3 ± 4.5% in the CM group. Furthermore, mean values of remaining xenogeneic BSM were 32.9 ± 6.3% in the BW group and 31.8 ± 8.8% in the CM group. Additionally, mean values for the connective tissue fractions were 39.2 ± 9.0% in the BW group and 37.9 ± 8.5% in the CM group. The trends represented by these histomorphometric outcomes did not indicate any meaningful differences between groups (Fig. 6).

## Discussion

The present study aimed to compare the sinus augmentation success in dependence on coverage of the bony window. Thus, the repositioning of the bony wall and closure of the bony window by a native collagen membrane following lateral access to the sinus cavity and implantation of a xenogeneic BSM were comparably analyzed using previously described clinical as well as histological and histomorphometrical methods [34, 36–40].

Furthermore, the present study reports on the duration of sinus augmentation surgery, lateral window preparation, and sinus mucosa preparation comparing the piezoelectric approach and conventional rotary instruments. According to our results, piezoelectric preparation tended to be consequently more time-efficient in every recorded parameter compared to rotary preparation, although differences were not significant. This result is contrary to general preconceptions among clinicians and the suggestion of Geminiani et al. [26]. The rate of sinus membrane perforations was comparable in both groups, i.e., 30% in the BW group compared to 35% in the CM group. These values are considerably higher compared to those of previous studies [26, 28, 29]. This might be explained by the fact that in the present study the minimum healing time following tooth extraction was 6 weeks and the majority of the sinus augmentation surgeries were performed 6–12 weeks following tooth removal. Consequently, incomplete healing of the Schneiderian membrane, as well as unlevelled interradicular septa, might have contributed to the relatively high prevalence of perforations. Nevertheless, none of the perforations exceeded 5 mm and could be covered by a collagen membrane, resulting in treatment success in each case. The cases with sinus membrane perforations showed comparable outcomes with regard to quality and quantity of hard tissues at 6 months; 2 months after reentry, all inserted implants were successfully osseointegrated.

Additionally, the present study reports on the comparative evaluation of postoperative discomfort between the piezoelectric approach and conventional rotary instruments. VAS values yielded consequently more favorable, although statistically not significantly lower values during the first 3 days of healing in the BW group. Furthermore, postoperative edema and hematoma were also lower in the BW group, although differences were not significant.

It should additionally be mentioned that the use of a barrier membrane also for the coverage of the osteotomy window may lead to better clinical results based on the fact that such kind of biomaterial protects the augmentation site against the exaggerated ingrowth of connective tissue and related disturbances of the bone regeneration process. This principle has manifoldly been documented in the case of jawbone reconstructions following the principle of Guided Bone Regeneration [41–43]. Interestingly, Barone and colleagues already showed in a clinical study comparing covered and non-covered lateral sinus windows that the use of a collagen membrane did not substantially increase the amount of vital bone over a period of 6 months but reduced the proliferation of the connective tissue [44]. Moreover, it is conceivable that the repositioning of the bony wall to the osteotomy site might lead to higher amounts of soft tissue within the sinus cavity due to faster and higher ingrowth into the sinus cavity through the piezosurgical cutting lines around the bony plate. This presumption may also be substantiated by the fact that soft or connective tissue is growing faster compared to bone tissue or bone matrix. To analyze this scientific issue, the histological and histomorphometrical analyses were additionally conducted in the present study. The histological analysis of the present study showed that an equal distribution of newly formed bone over the entire biopsy areas was observed in both study groups. The BSM was comparably integrated into the newly formed bone matrix covering the granule surfaces. Both results reveal the good osteoconductive properties of the xenogeneic BSM and did not reveal any differences between the two study groups. Also, no visible differences in the bone growth process or the characteristics of the bone matrix in the different biopsy areas were observed. Thus, no visible bone growth outgoing from the top of the biopsies originating from the reimplanted bony wall was observed. This result is contrary to a previous study conducted by Tawil et al., which described a bone growth also starting from repositioned bony walls in the lateral sinus wall using the same xenogeneic BSM [40]. However, both studies are not fully comparable as in the present study biopsies were retrieved from a crestal approach during guided implant osteotomy. In the aforementioned study by Tawil et al. biopsies from the lateral sinus wall including the implantation side of the bony wall or the collagen membrane were analyzed. Thus, it is conceivable that the bone growth outgoing from the reimplanted bony wall does not affect the bone growth process within the implant osteotomy area.

However, significant differences were measured interindividually in the BW group as the amount of connective tissue was significantly higher compared to the fraction of newly formed bone, while overall comparable results in terms of newly formed bone, residual BSM, and connective tissue formation were found in both study groups. Altogether, this result leads to the conclusion that the reimplanted bony wall seems to contribute to a slight shift of the tissue distribution towards higher amounts of connective tissue. This might be induced by the insertion of the bony window, which does not only include bone tissue but also connective tissue within the interspaces of the bone matrix that might have grown into the augmentation area. However, it did not affect the overall results even compared to the CM group and seems to be negligible as a sufficient amount of newly formed bone was detected. Other studies showed comparable results. For example, Johansson et al. compared the bone to implant contact using lateral sinus augmentation with autologous bone graft in combination with bony wall repositioning or collagen membrane coverage and application of autologous bone graft alone without using a bony wall or membrane [35]. No differences between the three groups could be shown, which generally suggests predictable treatment outcomes by the application of any of the aforementioned surgical approaches. In another study by Ohayon et al*.* with and without membrane coverage for sinus augmentation, the application of a membrane reduced the overall postoperative complication rate [22]. Based on these results, it could finally be concluded that both methods, i.e., the repositioning of the bony wall as well as the application of the native collagen membrane, lead to consistent and comparable levels of bone regeneration.

Moreover, these results seem mainly attributable to the choice of the BSM, which has shown to be a feasible material for osteoconductive bone growth as most of its surface areas were covered by newly formed bone. In this context, it has already been described in other clinical studies that the implanted xenogeneic BSM reliably contributes to bone growth within jawbone defects based on its natural characteristics [45–47]. The histomorphometrical analysis revealed around 30% of newly formed bone in both study groups without any statistically significant differences. Interestingly, the described amount of bone has also been found in different previous studies [48]. For example, Barbeck et al. showed in a combinatory preclinical and clinical study that the implantation of the xenogeneic BSM led to 31.63 ± 5.69% of newly formed bone also showing excellent osteoconductive properties [47]. Furthermore, Rothamel and colleagues analyzed the usability of the same BSM for one-stage and two-stage sinus floor elevation [49]. The results of this study showed good hard tissue regeneration of the lateral window of the sinus in all patients with proportions of newly formed bone within the graft between 25.8–49.6%. Thus, it can be concluded that the xenogeneic BSM reliably supports hard tissue regeneration after sinus floor elevation as shown by the different studies. Thereby, the healing properties of the analyzed BSM are comparable with another non-sintered xenogeneic BSM (Bio-Oss™, Geistlich Biomaterials, Wolhusen, Switzerland) that leads to similar clinical results showing the same excellent volume stability and integration into newly formed bone matrix within a six-month healing period [50].

Altogether, the clinical data of the present study lead to the conclusion that both methods seem to yield comparable results without any [dis-] advantages neither for the clinician nor the patient. Thus, it can be concluded that both methods lead to reliable clinical results. The histological results of the present study revealed that the BSM induced a very mild inflammatory tissue reaction mainly composed of macrophages and single multinucleated giant cells. Furthermore, no signs of biodegradation or phagocytosis have been found. In this context, it has already been reported in previous studies by Tawil et al. and Barbeck et al. that the analyzed high-temperature sintered bovine BSM is slowly resorbing. Additionally, different other clinical studies showed comparable results [40, 49]. For example, Rothamel et al*.* also revealed neither resorption nor dislocation of the granular bone substitute material. Also, the present study showed that the analyzed BSM is a suitable scaffold material for constant bone volume in the sinus cavity as the basis for dental implant placement.

Overall, maxillary sinus augmentation using the lateral approach represents a safe, reliable, and promising method for vertical hard tissue reconstruction of the posterior maxilla allowing for dental implant placement. The present study showed that bone regeneration takes place at a consistent and comparable level regardless of the methodology, i.e., bony wall repositioning or application of a native collagen membrane. In combination with the xenogeneic BSM, both methods lead to successful bone tissue growth within the sinus cavity as well as favorable clinical outcomes. The fact that piezoelectric window preparation was applied in the BW group and rotary instrumentation was utilized in the CM group is a limitation of the present study. Furthermore, the results obtained with the split-thickness flap design cannot be directly compared to other studies utilizing the standard full-thickness mucoperiosteal flap, since the split-thickness preparation may have had an influence on the healing of the bony window. Also, the distance between the lateral-medial walls may affect the healing of a sinus graft. Nevertheless, this factor was not taken into account during randomization and analysis.

## Conclusion

Within the limits of the present study, including the short-term follow-up, it can be concluded that maxillary sinus augmentation using the lateral approach in combination with a xenogeneic BSM and a native collagen membrane for lateral window coverage represents a reliable method for bone grafting. Piezosurgery with bony window repositioning delivers comparable outcomes without membrane coverage. The results of the present study show that the closure of the access window by means of the piezosurgically harvested autologous bony wall or the collagen membrane led to comparable bone augmentation results in combination with the BSM without any statistically significant clinical or histological differences between groups.
